# A rapid pupillometry protocol for clinical use: Effect of age and test-retest repeatability

**DOI:** 10.21203/rs.3.rs-7383219/v1

**Published:** 2025-09-19

**Authors:** Jason C. Park, J. Jason McAnany

**Affiliations:** University of Illinois Chicago; University of Illinois Chicago

## Abstract

**Purpose:**

Pupillometry is most commonly performed in laboratory settings using specialized, non-portable instruments that require lengthy test protocols. The purpose of this study was to develop and evaluate a rapid, clinically-applicable pupillometry protocol using a commercially available, portable, handheld instrument.

**Methods:**

Thirty-seven healthy individuals (ages 21- 61 years) participated in three experiments. In each experiment, the pupillary light reflex (PLR) was elicited by full-field, 500-ms chromatic flashes (470 nm and 621 nm; 12,000 Td). Experiment I evaluated the minimum dark adaptation (DA) time needed to achieve maximum PLRs. Experiment II determined the effect of age. Experiment III estimated PLR test-retest repeatability. For all experiments, baseline pupil size (BL; 1 sec before flash onset), maximum pupil constriction (MPC) following the flash, and post-illumination pupillary response (PIPR; median size 6 - 8 sec after flash offset) were quantified.

**Results:**

Experiment I showed that from 1 - 3 min of DA, BL and MPC increased slightly (0.27 mm and 5%, respectively), whereas the PIPR increased considerably (17%). The responses did not change appreciably after 3 min, therefore a 3-min DA period was used for Experiments II and III. Experiment II showed a trend for BL and MPC to decrease with age, but correlations with age were not statistically significant (all p > 0.05). PIPR was independent of age (r = −0.01; p = 0.96). Experiment III showed test-retest repeatability of approximately 1 mm for BL, and 10% for MPC and PIPR, indicating good repeatability.

**Conclusion:**

The proposed approach is useful for measuring the MPC and PIPR across a broad range of ages and baseline pupil sizes. Given the device portability and short test duration (approximately 5 minutes including DA), this approach has promising clinical utility.

## INTRODUCTION

Pupil size is adaptively modulated by the iris sphincter and dilator muscles to control the amount of light entering the eye. Although several factors affect pupil size, light stimulation is the primary driver and the response of the pupil to changes in illumination has been termed the pupillary light reflex (PLR). The PLR is largely mediated by intrinsically photosensitive retinal ganglion cells (ipRGCs) that also receive inputs from rod and cone photoreceptors [1–3]. Given that the PLR is dependent on the function of outer- and inner-retinal neurons, as well as afferent and efferent post-retina pathways, the PLR has become of interest in Ophthalmology and Neurology. Pupillometric assessments have been reported in patients with acquired [4–10] and inherited [11–16] ocular dysfunction. Beyond the retina, pupillometry has been applied to study central nervous system function in conditions including traumatic brain injury [17–19], Alzheimer’s disease [20–24], and Parkinson’s disease [25–29].

Traditionally, clinical assessment of the pupil has been performed qualitatively using a penlight (e.g., static measurement of pupil diameter under light-adapted conditions or with the “swinging flashlight test” to detect relative afferent pupillary defects). In recent decades, however, commercially available infrared videography systems have been developed that can provide quantitative measures of pupil size and reactivity. These infrared systems permit assessing pupil dynamics under both light- and dark-adapted conditions, which has become of interest in the clinical assessment of patients who have cone and rod photoreceptor dystrophies [12, 13, 30, 31]. The PLR elicited by a high-luminance, short-wavelength stimulus has been of particular interest, as this combination allows assessment of inner retinal function by stimulating ipRGCs. Specifically, the PLR elicited by a brief (≤ 1 s), high luminance, short-wavelength stimulus is characterized by a rapid, transient constriction that is thought to be rod- and cone-mediated [32–35]. The transient constriction is followed by a slow, sustained constriction that is often referred to as the post-illumination pupil response (PIPR) and is believed to be generated by melanopsin activation within ipRGCs [32, 34, 36].

Despite the promise of clinical pupillometry, the technique has largely been restricted to research applications in academic medical centers. Wider application has, in large part, been hampered by instrument cost, portability, and burdensome test lengths. There have been attempts to create portable pupillometers, including implementation using smart phones [37, 38] and other devices [39–42], but none are used routinely in clinical practice. These portable pupillometry devices differ considerably in their ease of use, data analysis capabilities, and ability to elicit PLRs mediated by the rod-, cone-, and melanopsin-pathways.

Recently, a portable, hand-held instrument for retinal electrophysiology (RETeval, LKC Technologies Inc., Gaithersburg, MD, USA) has been adapted to measure the PLR [16, 43, 44]. The instrument can produce stimuli that are high luminance and nearly full field in size, which are important features for eliciting a robust PIPR. The first report with this device [43] elicited pupil responses using low (50 cd/m^2^) and high (316 cd/m^2^) luminance flashes of blue light (470 nm) and the pupil size was recorded using built-in software, allowing for quantitative analyses. This protocol used only 3 minutes of dark-adaptation (DA), which expedited testing. Data from this small clinical study indicated that the maximum transient constriction differed between healthy control subjects (N = 5) and patients with optic neuropathy (N = 10); the PIPR was not reported. This report was followed by a second study [44] that obtained pupil responses from 32 healthy, young subjects (20–24 years of age) using 100 cd/m^2^ red and blue flashes of light. The DA period was 10 minutes; baseline pupil size and maximum transient constriction were reported. Robust pupil responses were elicited with the RETeval and the responses were in excellent agreement with a lab-based pupillometer (Iriscorder Dual, Hamamatsu Photonics, Hamamatsu, Japan). Most recently, the RETeval was used to examine pupil responses in patients with early onset high myopia (N = 14) and heathy emmetropic controls (N = 12) [16]. Following 10 min of DA, 250 cd/m^2^ red and blue flashes were presented and the baseline, maximum transient constriction, and PIPR were recorded. Reduced PIPRs were observed in myopic subjects who had MYP-26 gene variants.

All three studies show promise for the clinical application of portable pupillometry with the RETeval. However, the study protocols tended to be lengthy, data were obtained primarily from young subjects, and measures of test-retest repeatability were not performed. An additional limitation is that these studies used a constant flash luminance to elicit pupil constrictions, regardless of the subject’s baseline pupil size. This results in different amounts of light entering the eye, which may contribute to the inter-subject variability in these previous reports. Additionally, the studies were focused on the maximum transient constriction following the flash, with only one study reporting the PIPR. Thus, the purpose of the present study was to build on these previous reports to develop a rapid, clinically-applicable chromatic pupillometry protocol. In contrast to previous studies, stimuli were defined in Trolands, which accounted for differences in baseline pupil sizes among individuals, ensuring the same amount of light entered the eye of each subject. In Experiment I, the effect of DA duration was studied to define the minimum time needed to achieve maximum responses. In Experiment II, the effects of age on the transient and PIPR components of the PLR were determined. In Experiment III, test-retest repeatability of the transient and PIPR components was studied.

## METHODS

### Subjects

Thirty-seven healthy subjects were recruited from the University of Illinois Chicago, Department of Ophthalmology and Visual Sciences. The subjects were 20 to 61 years old (average 42 ± 13; 20 females). Subjects were excluded if there was a history of, or if they presented with, diabetes, glaucoma, optic neuropathy, or any other ophthalmic condition known to affect retinal or pupil function. Additionally, subjects with asymmetrically shaped pupils were excluded and no subject had a history of a neurologic event (cerebrovascular stroke or transient ischemic attack). The research followed the tenets of the Declaration of Helsinki and was approved by an institutional review board of the University of Illinois Chicago. All subjects provided written informed consent.

### Apparatus, stimuli, and procedure

Pupillometry was performed on the right eye of each subject using the RETeval device. The stimuli consisted of red (621 nm) and blue (470 nm) LED-generated flashes. The flash duration was 500 milliseconds and a constant stimulus flash retinal illuminance (12,000 Td) was generated by monitoring the subject’s pupil size using a built-in IR-sensitive camera and adjusting luminance accordingly. The IR camera has 0.08 mm/pixel resolution at the iris and a 28.3 Hz recording rate. Pupil diameters were exported using an extraction tool supplied by the manufacturer. The waveforms were processed using a custom script programmed in MATLAB R2021b (MathWorks Inc., Natick, MA), which allowed for semi-automated analysis as described elsewhere [7, 32]. For ease of processing, data were extracted and analyzed, but the output of the pupil metrics recorded in this study can now be obtained directly from the device’s display or from automatically generated accompanying PDF reports. The automatically generated reports were not available at the time the study was initiated, which necessitated the use of the off-line semi-automated approach. Subsequent comparison of the semi-automated analysis with the manufacturer’s automatically processed values found excellent consistency.

Measurement of the PLR elicited by the red flash always preceded the blue flash with an inter-stimulus interval (ISI) of at least 30 sec. Typically, a single PLR was obtained, but in cases of blinks or noise, the PLR measurement was repeated with an ISI of at least 60 sec. Baseline pupil size (BL) was defined as the median pupil size (mm) during the 1-sec preceding each stimulus onset, and each PLR sweep was normalized by the BL. Maximum pupil constriction (MPC) was defined as the difference between BL and the minimum pupil size after stimulus onset (normalized units). The post-illumination pupil response (PIPR) was defined as difference between the BL and the median pupil size at 6–8 sec after stimulus offset (normalized units).

The effect of DA duration was examined in Exp I in 13 subjects (44 ± 13.1 years; 7 female). For this experiment, PLRs were only elicited with the blue stimulus. The subjects were dark-adapted for randomly assigned duration of 1, 2, 3, or 4 min, then exposed to the blue flash. After recording, the subject was exposed to room light for least 2 min before the next randomly selected DA period began. The effect of age on BL, MPC, and PIPR was evaluated in Exp II in 37 subjects. Exp III examined test-retest repeatability using Bland-Altman analysis. A subset of 28 subjects (41.8 ± 11.3 years; 15 females) was tested twice using the same protocol developed in Exp II. The interval between two tests ranged from approximately 15 minutes to 292 days (mean test interval of 53.7 ± 105.8 days).

## RESULTS

**Figure 1:**
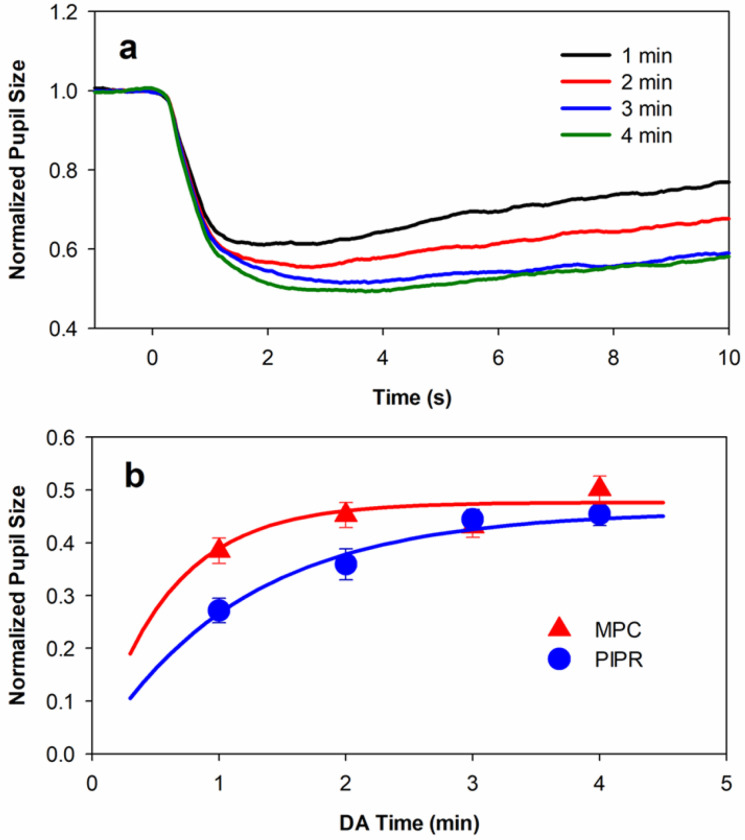
Panel a shows the mean normalized pupil traces following 1 min (black), 2 min (red), 3 min (blue), or 4 min (green) of dark-adaption. Panel b plots mean (±SEM) normalized MPC (red triangles) and PIPR (blue circles) as a function of the dark-adaptation time.

Figure 1a shows the mean normalized pupil traces following 1 min (black), 2 min (red), 3 min (blue), or 4 min (green) of DA. The PLRs were characterized by an initial transient constriction (“MPC;” rod- and cone-mediated) followed by a sustained constriction that persisted for several seconds after stimulus offset (“PIPR;” melanopsin-mediated). It is clear that both the MPC and PIPR increased from 1 to 3 min of DA, with relatively little change from 3 to 4 min. Figure 1b plots the mean (± SEM) MPC (red) and PIPR (blue) amplitudes as a function of DA duration. A two-parameter exponential growth to maximum function provide a good description of the data (solid lines). The MPC increased in amplitude from 1 to 2 min of DA and was essentially constant thereafter. The PIPR increased in amplitude from 1 to 3 min of DA, and was essentially constant thereafter. Of note, there was a clear difference in MPC and PIPR amplitude following 1 min DA, a small difference following 2 min DA, and little difference following 3 or 4 min DA. Similar values of MPC and PIPR are indicative of minimal return to baseline at 6–8 sec following the flash due to a robust melanopsin-mediated PIPR (apparent in the Fig. 1a). Based on these results, a 3-min DA period was used for Exp II and III.

**Figure 2:**
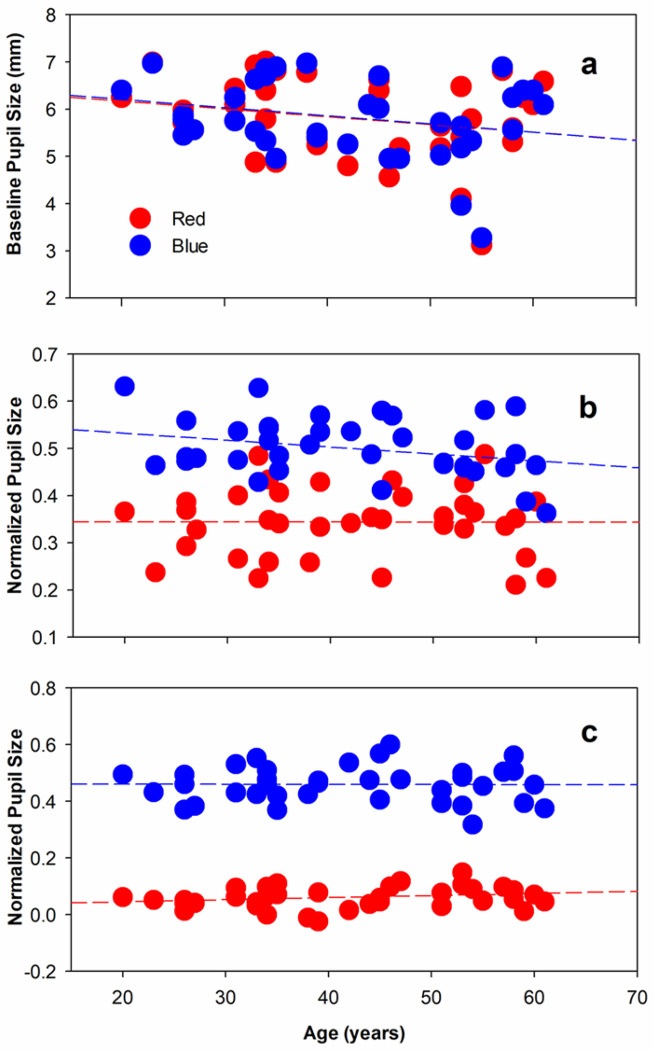
Baseline pupil diameter (a), MPC (b), and PIPR (c) are plotted as a function of the subject’s age. Data are shown for the long wavelength (red circles) and short wavelength (blue circles) stimuli and are fit with linear regression lines.

Figure 2 plots BL (a), MPC (b), and PIPR (c) as a function of the subject’s age; data are shown for red and blue stimuli and are fit with linear regression lines (dashed). BLs prior to the red and blue flashes were nearly identical for a given subject, with the red and blue regression lines essentially overlapping (Fig. 2a). There was a trend for BL to decrease with increasing age (red and blue MPC r >−0.23, p > 0.16), but this was not statistically significant. Figure 2b shows that the MPC elicited by the blue stimulus was larger than that elicited by the red stimulus. There was a trend for the blue MPC to decrease with increasing age (r = −0.32, p = 0.054), but this was not statistically significant; red MPC was independent of age (r = 0.003, p = 0.99). Figure 2c shows that the PIPR elicited by the red stimulus was negligible (mean red PIPR = 0.06) and there was no significant correlation with age (r = 0.26, p = 0.12). The blue stimulus elicited a large PIPR (mean blue PIPR = 0.46), but there was no significant correlation with age (r = −0.01, p = 0.96).

Previous work identified weak effects of sex on the PLR, with females having slightly larger MPC under dark-adapted conditions, compared to males [45, 46]. A possible effect of sex on BL, MPC, and PIPR was evaluated in the present data set. Two-way ANOVAs were conducted with stimulus wavelength and subject sex as main effects. ANOVA indicated no significant effect of sex on BL, MPC, or PIPR (F < 0.64, p > 0.43) and no interaction between sex and stimulus wavelength for any measure (F < 3.34, p > 0.07).

**Figure 3:**
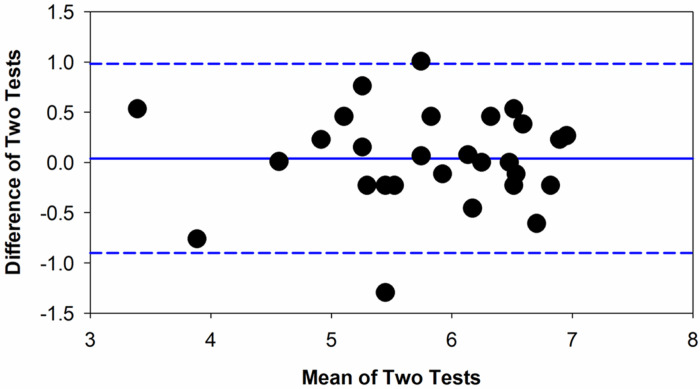
Test-retest repeatability for the baseline pupil size is shown in the form of Bland-Altman plot. Each circle represents the difference between the two tests as a function of the mean of the two tests. The solid line represents the mean difference between tests for the 27 subjects and the dashed lines present the 95% limits of repeatability.

Figure 3 plots the difference in BL for the two repeat tests as a function of the mean of the two tests in the form of a Bland-Altman plot. Data are only shown for the BL preceding the red stimulus. The mean BL difference for the two tests was 0.04 mm (0.7%; solid blue line) and the upper and lower limits of repeatability (± 95%; dashed lines) were + 0.98 mm and − 0.90 mm, respectively. The difference between the two tests was not significantly correlated with the overall BL size (r = 0.01, p = 0.96).

**Figure 4:**
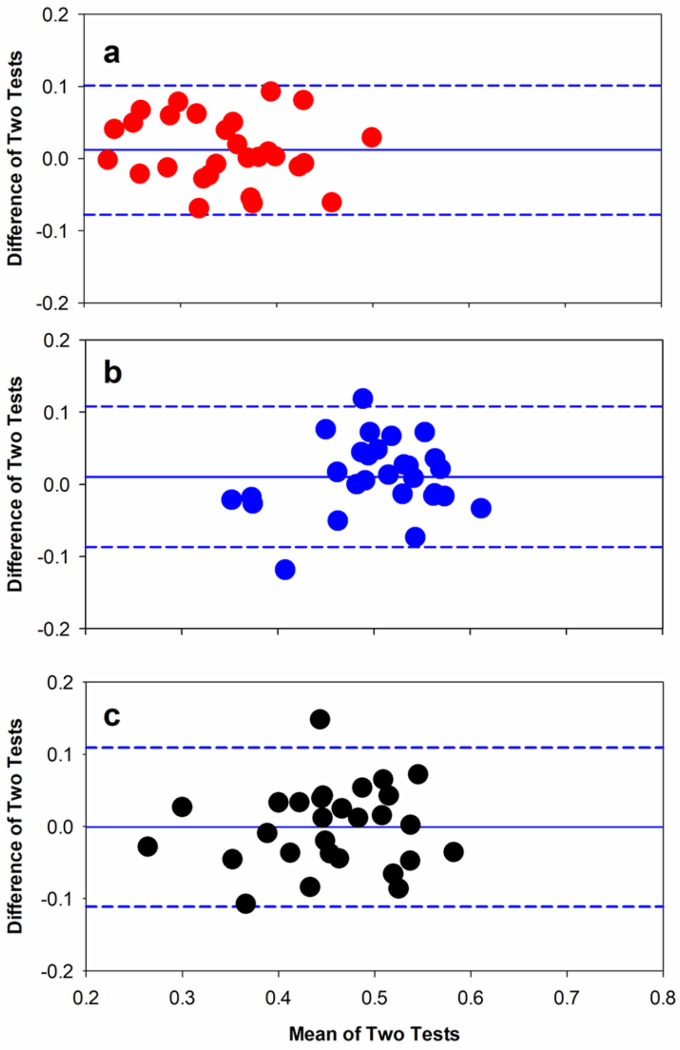
Test-retest repeatability for the MPC elicited by the red (a) and blue (b) stimuli shown in the form of Bland-Altman plot. Panel C shows test-retest repeatability for the PIPR (blue). All other conventions are as in Fig. 3.

Figure 4a and 4b represent repeat measurements of the red and blue MPC in the same Bland-Altman format as Fig. 3. Figure 4a shows the mean difference between tests for the red MPC was 0.01, with an upper limit of repeatability of 0.10 and a lower limit of −0.08. Figure 4b shows the mean difference between tests for the blue MPC was 0.01, with an upper limit of repeatability of 0.10 and a lower limit of −0.09. Figure 4c shows the mean difference between the PIPR measurements for the blue stimulus. The mean difference between tests was − 0.001, with upper and lower limits of repeatability of + 0.11 and − 0.11, respectively. The difference between the two tests was not significantly correlated with the overall MPC or PIPR size (all p > 0.37)

## DISCUSSION

The purpose of this study was to develop and evaluate a rapid, clinically-applicable pupillometry protocol using a commercially available, portable, handheld instrument. The primary findings were 1) a 3-min DA period is sufficient to elicit robust MPCs and PIPRs; 2) there is no statistically significant relationship between age and BL, MPC, or PIPR in our sample of subjects across the ages of 26–61 years; 3) test-retest repeatability was good, with differences within approximately 10% observed. The total test time, including DA, for one eye was approximately 5 min.

DA periods of 10 to 15 min are commonly employed in pupillometry studies [32, 34, 47]. Although less demanding than the ISCEV standard 20 min DA for full-field electroretinography [48], DA remains a burden in clinical testing. Data of the present study show that 3 min is sufficient to elicit large MPCs and PIPRs in healthy, visually-normal individuals. This finding is consistent with the conclusion of Bindiganavale [43] who reported maximum pupillary constrictions following three minutes of DA. We observed minimal differences in pupil response between 3 and 4 min, and therefore used 3 min DA to expedite testing. Although the stimulus parameters selected for this study elicited large MPCs and PIPRs after only 3 min of DA, a 3-min DA period may not elicit maximum MPCs and PIPRs for other stimuli. For example, Wang et al. [47] reported that the PIPR increased over a time-course of approximately 20 min of DA for a 100 cd/m^2^ flash (1 sec; 465 nm). It is also important to note that that present findings were obtained only from healthy, visually-normal subjects and that DA dynamics may differ in patient populations.

Somewhat surprisingly, there was relatively little effect of age on the pupil metrics that were assessed in the present study. BL decreased by 0.17 mm with each increasing decade of age, but this relationship did not achieve statistical significance. “Senile miosis” is a well-known effect of aging [49–52]. It is possible that the effect of age on BL may have been more apparent in the present dataset if a larger number of subjects older than 60 years of age were included. Studies reporting the effect of age on the human PIPR have been inconsistent [53–55]. Age-related loss of ipRGC subtypes has been reported in the human retina [56], but the loss appears most prominently after the age of 70. Rather than loss of ipRGCs, reduced blue light sensitivity due to cataract formation may be a more influential factor to consider when interpreting chromatic pupillometry results across age.

Test-retest repeatability in the present sample of subjects was good, with differences less than approximately 10% observed, defined by Bland-Altman analyses. Herbst et al. [57] reported intraclass coefficients (ICCs) for maximum constrictions elicited by red and blue flashes measured with a custom pupilometer to be 0.7 to 0.8, which is similar to our MPC ICC value of 0.71. Li et al. [58] reported ICCs of 0.84 to 0.94 for the PIPR elicited by full-field blue flashes, which is similar to our PIPR ICC of 0.97. Of note, the present study is underpowered to assess test-retest repeatability within a narrow confidence interval. To define repeatability within a 10% confidence interval, for example, 190 subjects would be needed. Our sample size of 27 subjects provides a much larger confidence interval of 27%. Nevertheless, the data provided herein represents a first step toward a potential standardized approach to rapid portable pupillometry.

In summary, we provide evidence that a short-duration, clinically applicable, chromatic pupillometry test can be used to assess function in healthy individuals. The test is administered quickly (approximately 5 min for one eye), is well tolerated, and repeatable.
